# Crystal structure of 4-(6-bromo-4-oxo-4*H*-chromen-3-yl)-2-methyl­amino-3-nitro­pyrano[3,2-*c*]chromen-5(4*H*)-one chloro­form monosolvate

**DOI:** 10.1107/S2056989015014553

**Published:** 2015-08-06

**Authors:** Rajamani Raja, Subramani Kandhasamy, Paramasivam T. Perumal, A. SubbiahPandi

**Affiliations:** a Department of Physics, Presidency College (Autonomous), Chennai 600 005, India; b Organic Chemistry Division, CSIR Central Leather Research Institute, Chennai 600 020, India

**Keywords:** crystal structure, chromenone, hydrogen bonding

## Abstract

In the title compound, C_22_H_13_BrN_2_O_7_·CHCl_3_, the pyran ring adopts a shallow sofa conformation with the C atom bearing the bromo­chromene system as the flap [deviation = 0.291 (3) Å]. The dihedral angle between the pyran fused-ring system (all atoms; r.m.s. deviation = 0.032 Å) and the bromo­chromene ring system (r.m.s. deviation = 0.027 Å) is 87.56 (9)°. An intra­molecular N—H⋯O hydrogen bond closes an *S*(6) ring. The Cl atoms of the solvent mol­ecule are disordered over two sets of sites in a 0.515 (6):0.485 (6) ratio. In the crystal, inversion dimers linked by pairs of N—H⋯O hydrogen bonds generate *R*_2_^2^(12) loops. The packing also features C—H⋯O and very weak π–π [centroid–centroid separation = 3.960 (2) Å] inter­actions, which link the dimers into a three-dimensional network.

## Related literature   

For background to chromene derivatives, see: Ercole *et al.* (2009 ▸); Geen *et al.* (1996 ▸) Khan *et al.* (2010 ▸); Raj *et al.* (2010 ▸). For a related structure, see: Raja *et al.* (2015 ▸).
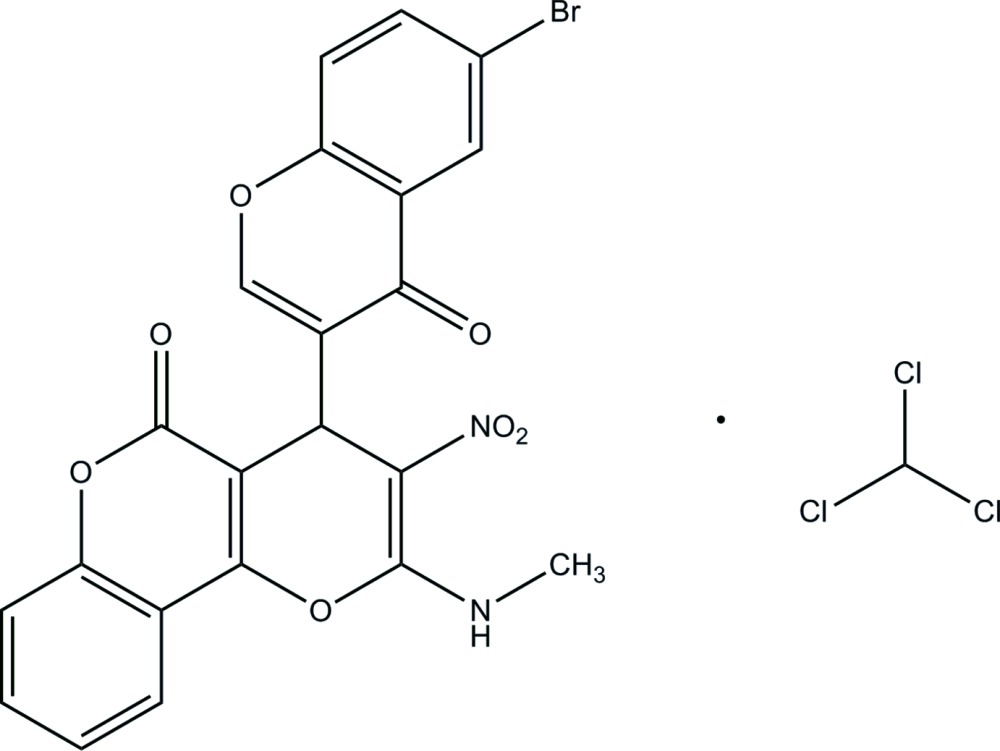


## Experimental   

### Crystal data  

C_22_H_13_BrN_2_O_7_·CHCl_3_
*M* *_r_* = 616.62
Triclinic, 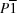

*a* = 9.8816 (2) Å
*b* = 11.9237 (3) Å
*c* = 12.0616 (3) Å
α = 80.804 (1)°
β = 68.422 (1)°
γ = 70.735 (1)°
*V* = 1246.36 (5) Å^3^
*Z* = 2
Mo *K*α radiation
μ = 2.02 mm^−1^
*T* = 293 K
0.35 × 0.30 × 0.25 mm

### Data collection  

Bruker SMART APEXII CCD diffractometer
Absorption correction: multi-scan (*SADABS*; Bruker, 2008 ▸) *T*_min_ = 0.539, *T*_max_ = 0.632
17277 measured reflections
4389 independent reflections
3672 reflections with *I* > 2σ(*I*)
*R*_int_ = 0.019

### Refinement  

*R*[*F* ^2^ > 2σ(*F* ^2^)] = 0.043
*wR*(*F* ^2^) = 0.120
*S* = 1.04
4389 reflections
353 parameters
114 restraints
H atoms treated by a mixture of independent and constrained refinement
Δρ_max_ = 0.63 e Å^−3^
Δρ_min_ = −0.53 e Å^−3^

### 

Data collection: *APEX2* (Bruker, 2008 ▸); cell refinement: *SAINT* (Bruker, 2008 ▸); data reduction: *SAINT*; program(s) used to solve structure: *SHELXS97* (Sheldrick, 2008 ▸); program(s) used to refine structure: *SHELXL97* (Sheldrick, 2008 ▸); molecular graphics: *ORTEP-3 for Windows* (Farrugia, 2012 ▸); software used to prepare material for publication: *SHELXL97* and *PLATON* (Spek, 2009 ▸).

## Supplementary Material

Crystal structure: contains datablock(s) global, I. DOI: 10.1107/S2056989015014553/hb7473sup1.cif

Structure factors: contains datablock(s) I. DOI: 10.1107/S2056989015014553/hb7473Isup2.hkl

Supporting information file. DOI: 10.1107/S2056989015014553/hb7473Isup3.cml

**Fig. 1.**
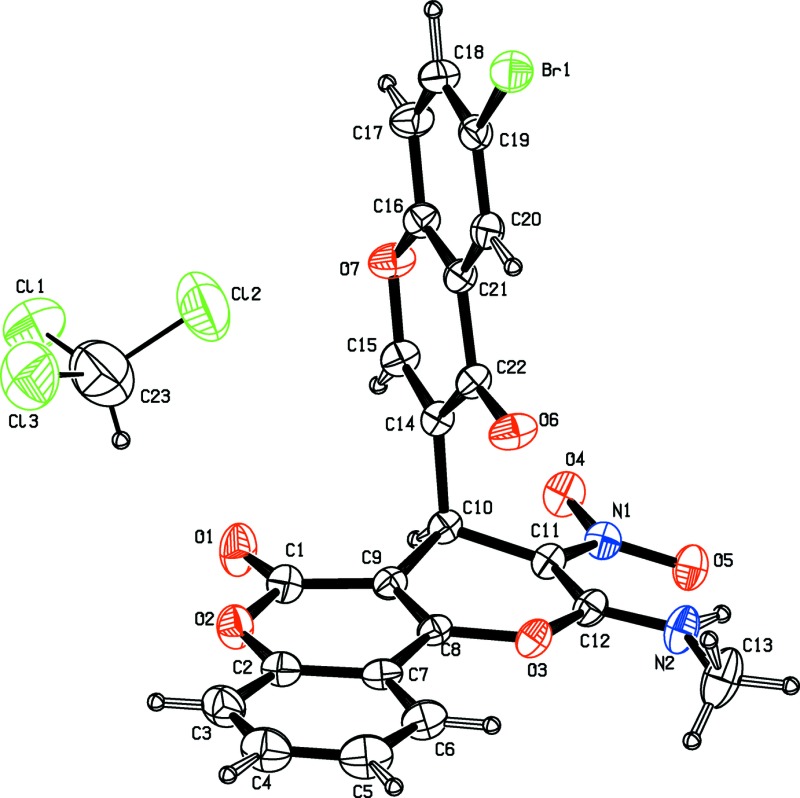
The molecular structure of the title molecule, with displacement ellipsoids drawn at 30% probability level. The intramolecular hydrogen bond, which generates an S(6) ring motif, is shown as a dashed line.

S . DOI: 10.1107/S2056989015014553/hb7473fig1.tif
The mol­ecular structure of the title mol­ecule, with displacement ellipsoids drawn at 30% probability level. The intra­molecular hydrogen bond, which generates an *S*(6) ring motif, is shown as a dashed line.

**Fig. 2.**
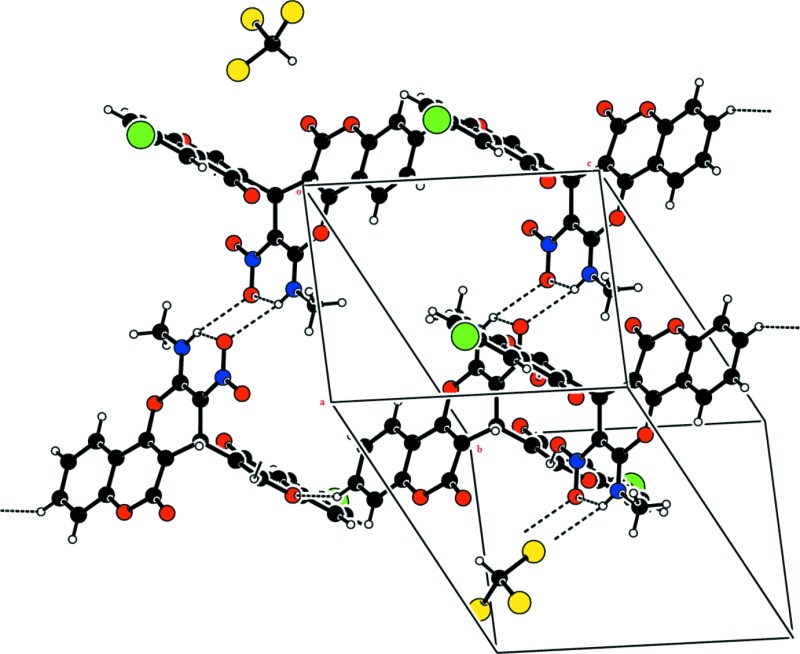
Packing diagram showing the chain motif R22(12) along the [100] direction.

. DOI: 10.1107/S2056989015014553/hb7473fig2.tif
Packing diagram showing the chain motif 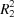
(12) along the [100] direction.

CCDC reference: 1416576

Additional supporting information:  crystallographic information; 3D view; checkCIF report

## Figures and Tables

**Table 1:** Hydrogen-bond geometry (, )

*D*H*A*	*D*H	H*A*	*D* *A*	*D*H*A*
N2H2O5	0.86	2.00	2.622(5)	128
N2H2O5^i^	0.86	2.37	3.063(5)	138
C4H4O7^ii^	0.93	2.59	3.383(6)	144
C15H15O4^iii^	0.93	2.36	3.221(4)	153

Symmetry codes: (i) 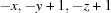; (ii) 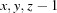; (iii) 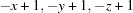.

## supplementary crystallographic information

### S1. Comment

Chromene derivatives are heterocyclic compounds that have a variety of industrial, biological and chemical synthesis applications (Geen *et al.*, 1996; Ercole *et al.*, 2009). They exhibit a number of pharmacological activities such as anti-HIV, anti-inflammatory, anti-bacterial, anti-allergic, anti-cancer, *etc*. (Khan *et al.*, 2010; Raj *et al.*, 2010). Against this background an X-ray diffraction study of the title compound and its structural aspects are presented herein.

The asymmetric unit of the title compound is shown in Fig.1. The six-membered central pyran ring is very similar to a screw boat conformation as evidenced by the puckering parameters q_2_ = 0.204 (4) Å, θ = 112.7 (11) and φ = 6.7 (12)°, respectively. The atoms C10 and O3 are deviating from the mean plane of C8—C9—C11—C12 by -0.266 and -0.644 Å, respectively. The chromene ring (O2/C1—C9) and (O7/C14—C22) are almost planar and normal to one another with a dihedral angle of 88.20 (2)° between their mean planes. The nitro group is bonded to the pyran ring at CC with the torsion angle C12—C11—N1—O5 0f 3.5 (5)°, indicating a (+) *syn*-periplanar conformation for this group. The chromene ring attached to the pyran ring at C10 with torsion angle C11—C10—C14—C15 of 117.6 (4)°, indicating a (+) anti-clinal conformation for this group. The title compound exhibits structural similarities with already reported related structure (Raja *et al.*, 2015).

In the crystal structure, the molecules are linked to form an infinite chain along [100], through N2—H···O5 hydrogen bonds, generating graph set motifs *R*_2_^2^(12) (Fig.2). In addition, there is a N—H···O intramolecular interaction.

### S2. Experimental

4-Hydroxycoumarin (0.81 g, 5 mmol), 6-bromo-4-oxo-4*H*-chromene-3-carbaldehyde (0.78 g, 5 mmol) and NMSM (0.74 g, 5 mmol) were mixed in ethanol at room temperature (3 h) in the presence of TEA (triethylamine 0.1 eq), as a catalyst. Upon completion of the reaction, the mixture was filtered, and washed with ethanol to obtained desired white product in 93% yield. Colourless blocks of the title compound were recrystallised from chloroform solution.

### S3. Refinement

N and C-bound H atoms were positioned geometrically (C–H = 0.93–0.98 Å) and allowed to ride on their parent atoms, with *U*_iso_(H) = 1.5*U*_eq_(C) for methyl H atoms and 1.2*U*_eq_(C) for all other H atoms.

### Crystal data

**Table:**

C_22_H_13_BrN_2_O_7_·CHCl_3_	*Z* = 2
*M**_r_* = 616.62	*F*(000) = 616
Triclinic, *P*1	*D*_x_ = 1.643 Mg m^−^^3^
Hall symbol: -P 1	Mo *K*α radiation, λ = 0.71073 Å
*a* = 9.8816 (2) Å	Cell parameters from 3672 reflections
*b* = 11.9237 (3) Å	θ = 1.8–25.0°
*c* = 12.0616 (3) Å	µ = 2.02 mm^−^^1^
α = 80.804 (1)°	*T* = 293 K
β = 68.422 (1)°	Colourless, block
γ = 70.735 (1)°	0.35 × 0.30 × 0.25 mm
*V* = 1246.36 (5) Å^3^	

### Data collection

**Table:**

Bruker SMART APEXII CCD diffractometer	4389 independent reflections
Radiation source: fine-focus sealed tube	3672 reflections with *I* > 2σ(*I*)
Graphite monochromator	*R*_int_ = 0.019
ω and φ scans	θ_max_ = 25.0°, θ_min_ = 1.8°
Absorption correction: multi-scan (*SADABS*; Bruker, 2008)	*h* = −11→10
*T*_min_ = 0.539, *T*_max_ = 0.632	*k* = −14→14
17277 measured reflections	*l* = −14→14

### Refinement

**Table:**

Refinement on *F*^2^	Primary atom site location: structure-invariant direct methods
Least-squares matrix: full	Secondary atom site location: difference Fourier map
*R*[*F*^2^ > 2σ(*F*^2^)] = 0.043	Hydrogen site location: inferred from neighbouring sites
*wR*(*F*^2^) = 0.120	H atoms treated by a mixture of independent and constrained refinement
*S* = 1.04	*w* = 1/[σ^2^(*F*_o_^2^) + (0.0626*P*)^2^ + 1.1978*P*] where *P* = (*F*_o_^2^ + 2*F*_c_^2^)/3
4389 reflections	(Δ/σ)_max_ < 0.001
353 parameters	Δρ_max_ = 0.63 e Å^−^^3^
114 restraints	Δρ_min_ = −0.53 e Å^−^^3^

### Special details

**Table:**

Geometry. All e.s.d.'s (except the e.s.d. in the dihedral angle between two l.s. planes) are estimated using the full covariance matrix. The cell e.s.d.'s are taken into account individually in the estimation of e.s.d.'s in distances, angles and torsion angles; correlations between e.s.d.'s in cell parameters are only used when they are defined by crystal symmetry. An approximate (isotropic) treatment of cell e.s.d.'s is used for estimating e.s.d.'s involving l.s. planes.
Refinement. Refinement of *F*^2^ against ALL reflections. The weighted *R*-factor *wR* and goodness of fit *S* are based on *F*^2^, conventional *R*-factors *R* are based on *F*, with *F* set to zero for negative *F*^2^. The threshold expression of *F*^2^ > σ(*F*^2^) is used only for calculating *R*-factors(gt) *etc*. and is not relevant to the choice of reflections for refinement. *R*-factors based on *F*^2^ are statistically about twice as large as those based on *F*, and *R*- factors based on ALL data will be even larger.

### Fractional atomic coordinates and isotropic or equivalent isotropic displacement parameters (Å^2^)

**Table:**

	*x*	*y*	*z*	*U*_iso_*/*U*_eq_	Occ. (<1)
C1	0.6175 (4)	0.6474 (3)	0.0736 (4)	0.0442 (9)	
C2	0.5491 (5)	0.6922 (3)	−0.1038 (3)	0.0447 (9)	
C3	0.5920 (6)	0.7284 (4)	−0.2223 (4)	0.0592 (11)	
H3	0.6853	0.7436	−0.2601	0.071*	
C4	0.4958 (6)	0.7418 (5)	−0.2839 (4)	0.0678 (13)	
H4	0.5237	0.7667	−0.3640	0.081*	
C5	0.3578 (6)	0.7189 (5)	−0.2288 (4)	0.0675 (13)	
H5	0.2936	0.7283	−0.2721	0.081*	
C6	0.3140 (5)	0.6820 (4)	−0.1098 (4)	0.0545 (11)	
H6	0.2211	0.6660	−0.0732	0.065*	
C7	0.4101 (4)	0.6691 (3)	−0.0449 (3)	0.0402 (8)	
C8	0.3759 (4)	0.6353 (3)	0.0800 (3)	0.0351 (8)	
C9	0.4701 (4)	0.6296 (3)	0.1389 (3)	0.0355 (8)	
C10	0.4260 (4)	0.6090 (3)	0.2719 (3)	0.0341 (8)	
H10	0.5132	0.5529	0.2910	0.041*	
C11	0.2968 (4)	0.5549 (3)	0.3146 (3)	0.0362 (8)	
C12	0.2024 (4)	0.5667 (3)	0.2490 (3)	0.0395 (8)	
C13	−0.0189 (6)	0.5523 (5)	0.2111 (5)	0.0758 (16)	
H13A	−0.1049	0.5241	0.2563	0.114*	
H13B	0.0393	0.5076	0.1405	0.114*	
H13C	−0.0538	0.6349	0.1888	0.114*	
C14	0.3830 (4)	0.7256 (3)	0.3303 (3)	0.0344 (8)	
C15	0.4574 (4)	0.7360 (3)	0.3991 (3)	0.0415 (8)	
H15	0.5365	0.6704	0.4080	0.050*	
C16	0.3129 (4)	0.9334 (3)	0.4428 (3)	0.0406 (8)	
C17	0.2842 (5)	1.0337 (4)	0.5026 (4)	0.0530 (10)	
H17	0.3397	1.0319	0.5509	0.064*	
C18	0.1736 (5)	1.1350 (4)	0.4898 (4)	0.0528 (10)	
H18	0.1529	1.2026	0.5296	0.063*	
C19	0.0923 (4)	1.1360 (3)	0.4165 (3)	0.0423 (9)	
C20	0.1176 (4)	1.0372 (3)	0.3593 (3)	0.0396 (8)	
H20	0.0607	1.0391	0.3120	0.047*	
C21	0.2297 (4)	0.9331 (3)	0.3724 (3)	0.0362 (8)	
C22	0.2577 (4)	0.8247 (3)	0.3136 (3)	0.0368 (8)	
N1	0.2703 (4)	0.4975 (3)	0.4251 (3)	0.0405 (7)	
N2	0.0763 (4)	0.5375 (3)	0.2832 (3)	0.0519 (9)	
H2	0.0471	0.5069	0.3543	0.062*	
O1	0.7135 (3)	0.6371 (3)	0.1160 (3)	0.0648 (9)	
O2	0.6502 (3)	0.6786 (3)	−0.0456 (2)	0.0533 (7)	
O3	0.2394 (3)	0.6112 (2)	0.1340 (2)	0.0408 (6)	
O4	0.3527 (3)	0.4944 (2)	0.4837 (2)	0.0506 (7)	
O5	0.1646 (3)	0.4491 (3)	0.4673 (3)	0.0514 (7)	
O6	0.1798 (3)	0.8194 (2)	0.2569 (3)	0.0525 (7)	
O7	0.4263 (3)	0.8351 (2)	0.4569 (2)	0.0498 (7)	
Br1	−0.05894 (5)	1.27713 (4)	0.39770 (4)	0.05830 (19)	
C23	0.7698 (6)	0.9052 (8)	0.0829 (5)	0.146 (3)	
H23A	0.7777	0.8283	0.0580	0.175*	0.515 (6)
H23B	0.7592	0.8259	0.1122	0.175*	0.485 (6)
Cl1	0.9143 (5)	0.8826 (4)	0.1496 (4)	0.1075 (15)	0.515 (6)
Cl2	0.5958 (5)	0.9568 (5)	0.1863 (5)	0.140 (2)	0.515 (6)
Cl3	0.8013 (6)	0.9975 (5)	−0.0393 (3)	0.131 (2)	0.515 (6)
Cl1'	0.8251 (14)	0.9193 (11)	0.1864 (7)	0.260 (6)	0.485 (6)
Cl2'	0.5804 (9)	0.9669 (10)	0.0975 (13)	0.317 (8)	0.485 (6)
Cl3'	0.8819 (8)	0.8620 (7)	−0.0529 (4)	0.174 (3)	0.485 (6)

### Atomic displacement parameters (Å^2^)

**Table:**

	*U*^11^	*U*^22^	*U*^33^	*U*^12^	*U*^13^	*U*^23^
C1	0.043 (2)	0.044 (2)	0.046 (2)	−0.0120 (17)	−0.0174 (18)	0.0013 (17)
C2	0.052 (2)	0.040 (2)	0.042 (2)	−0.0108 (18)	−0.0176 (18)	−0.0028 (16)
C3	0.067 (3)	0.062 (3)	0.044 (2)	−0.019 (2)	−0.014 (2)	−0.001 (2)
C4	0.087 (4)	0.071 (3)	0.044 (3)	−0.020 (3)	−0.027 (3)	0.003 (2)
C5	0.086 (4)	0.074 (3)	0.055 (3)	−0.018 (3)	−0.046 (3)	0.001 (2)
C6	0.065 (3)	0.058 (3)	0.052 (3)	−0.017 (2)	−0.034 (2)	−0.001 (2)
C7	0.051 (2)	0.0308 (18)	0.041 (2)	−0.0077 (16)	−0.0213 (18)	−0.0040 (15)
C8	0.0380 (19)	0.0290 (17)	0.041 (2)	−0.0087 (15)	−0.0175 (16)	−0.0025 (14)
C9	0.0356 (19)	0.0303 (17)	0.042 (2)	−0.0076 (15)	−0.0170 (16)	−0.0012 (14)
C10	0.0339 (18)	0.0322 (17)	0.0410 (19)	−0.0075 (14)	−0.0215 (15)	0.0021 (14)
C11	0.0377 (19)	0.0330 (18)	0.041 (2)	−0.0105 (15)	−0.0187 (16)	0.0043 (15)
C12	0.041 (2)	0.0343 (18)	0.049 (2)	−0.0137 (16)	−0.0212 (17)	0.0053 (16)
C13	0.066 (3)	0.096 (4)	0.094 (4)	−0.045 (3)	−0.055 (3)	0.032 (3)
C14	0.0360 (19)	0.0353 (18)	0.0355 (18)	−0.0116 (15)	−0.0174 (15)	0.0042 (14)
C15	0.045 (2)	0.0393 (19)	0.045 (2)	−0.0096 (17)	−0.0255 (18)	0.0016 (16)
C16	0.045 (2)	0.043 (2)	0.038 (2)	−0.0138 (17)	−0.0176 (17)	−0.0001 (16)
C17	0.061 (3)	0.058 (3)	0.051 (2)	−0.018 (2)	−0.028 (2)	−0.010 (2)
C18	0.061 (3)	0.046 (2)	0.053 (2)	−0.019 (2)	−0.014 (2)	−0.0124 (19)
C19	0.041 (2)	0.0357 (19)	0.044 (2)	−0.0124 (16)	−0.0075 (17)	−0.0007 (16)
C20	0.039 (2)	0.039 (2)	0.041 (2)	−0.0134 (16)	−0.0146 (16)	0.0035 (16)
C21	0.0376 (19)	0.0354 (18)	0.0376 (19)	−0.0126 (15)	−0.0144 (16)	0.0011 (15)
C22	0.039 (2)	0.0373 (19)	0.0399 (19)	−0.0118 (16)	−0.0210 (16)	0.0021 (15)
N1	0.0407 (18)	0.0347 (16)	0.0451 (18)	−0.0081 (14)	−0.0186 (15)	0.0046 (13)
N2	0.048 (2)	0.062 (2)	0.059 (2)	−0.0286 (17)	−0.0291 (17)	0.0172 (17)
O1	0.0446 (17)	0.098 (3)	0.0627 (19)	−0.0300 (17)	−0.0268 (15)	0.0082 (17)
O2	0.0475 (16)	0.0693 (19)	0.0447 (16)	−0.0225 (14)	−0.0151 (13)	0.0028 (14)
O3	0.0430 (15)	0.0440 (14)	0.0461 (15)	−0.0173 (12)	−0.0265 (12)	0.0064 (11)
O4	0.0554 (17)	0.0539 (17)	0.0505 (16)	−0.0164 (14)	−0.0336 (14)	0.0148 (13)
O5	0.0497 (16)	0.0521 (16)	0.0548 (17)	−0.0239 (14)	−0.0188 (13)	0.0133 (13)
O6	0.0569 (17)	0.0432 (15)	0.0710 (19)	−0.0030 (13)	−0.0450 (16)	−0.0083 (13)
O7	0.0592 (18)	0.0485 (16)	0.0545 (17)	−0.0080 (13)	−0.0388 (14)	−0.0067 (13)
Br1	0.0560 (3)	0.0359 (2)	0.0753 (3)	−0.00750 (19)	−0.0191 (2)	−0.00208 (19)
C23	0.127 (7)	0.178 (8)	0.117 (6)	−0.035 (6)	−0.027 (5)	−0.024 (6)
Cl1	0.125 (3)	0.100 (3)	0.118 (4)	−0.038 (2)	−0.061 (3)	−0.005 (2)
Cl2	0.107 (3)	0.123 (4)	0.134 (4)	−0.031 (3)	0.019 (3)	−0.002 (3)
Cl3	0.157 (4)	0.152 (5)	0.084 (2)	−0.078 (3)	−0.028 (2)	0.028 (2)
Cl1'	0.426 (17)	0.233 (10)	0.126 (5)	−0.091 (11)	−0.083 (8)	−0.056 (6)
Cl2'	0.257 (11)	0.154 (7)	0.425 (19)	0.054 (7)	−0.078 (12)	−0.041 (11)
Cl3'	0.214 (7)	0.215 (8)	0.098 (3)	−0.102 (6)	−0.032 (4)	0.009 (4)

### Geometric parameters (Å, º)

**Table:**

C1—O1	1.199 (5)	C14—C15	1.337 (5)
C1—O2	1.370 (5)	C14—C22	1.453 (5)
C1—C9	1.446 (5)	C15—O7	1.358 (5)
C2—C3	1.374 (6)	C15—H15	0.9300
C2—O2	1.374 (5)	C16—O7	1.367 (5)
C2—C7	1.391 (6)	C16—C21	1.383 (5)
C3—C4	1.365 (7)	C16—C17	1.390 (6)
C3—H3	0.9300	C17—C18	1.368 (6)
C4—C5	1.377 (7)	C17—H17	0.9300
C4—H4	0.9300	C18—C19	1.392 (6)
C5—C6	1.381 (7)	C18—H18	0.9300
C5—H5	0.9300	C19—C20	1.366 (5)
C6—C7	1.397 (5)	C19—Br1	1.893 (4)
C6—H6	0.9300	C20—C21	1.397 (5)
C7—C8	1.437 (5)	C20—H20	0.9300
C8—C9	1.344 (5)	C21—C22	1.470 (5)
C8—O3	1.369 (4)	C22—O6	1.223 (4)
C9—C10	1.501 (5)	N1—O4	1.248 (4)
C10—C11	1.505 (5)	N1—O5	1.264 (4)
C10—C14	1.521 (5)	N2—H2	0.8600
C10—H10	0.9800	C23—Cl1'	1.587 (7)
C11—N1	1.372 (5)	C23—Cl3'	1.653 (6)
C11—C12	1.391 (5)	C23—Cl3	1.688 (7)
C12—N2	1.307 (5)	C23—Cl2	1.691 (6)
C12—O3	1.364 (4)	C23—Cl2'	1.721 (7)
C13—N2	1.454 (5)	C23—Cl1	1.812 (6)
C13—H13A	0.9600	C23—H23A	0.9800
C13—H13B	0.9600	C23—H23B	0.9800
C13—H13C	0.9600		
			
O1—C1—O2	117.3 (4)	C18—C17—H17	120.3
O1—C1—C9	124.9 (4)	C16—C17—H17	120.3
O2—C1—C9	117.8 (3)	C17—C18—C19	119.4 (4)
C3—C2—O2	117.1 (4)	C17—C18—H18	120.3
C3—C2—C7	121.8 (4)	C19—C18—H18	120.3
O2—C2—C7	121.1 (3)	C20—C19—C18	121.6 (4)
C4—C3—C2	119.0 (5)	C20—C19—Br1	119.3 (3)
C4—C3—H3	120.5	C18—C19—Br1	119.1 (3)
C2—C3—H3	120.5	C19—C20—C21	119.5 (3)
C3—C4—C5	120.9 (4)	C19—C20—H20	120.3
C3—C4—H4	119.6	C21—C20—H20	120.3
C5—C4—H4	119.6	C16—C21—C20	118.8 (3)
C4—C5—C6	120.5 (4)	C16—C21—C22	120.5 (3)
C4—C5—H5	119.8	C20—C21—C22	120.7 (3)
C6—C5—H5	119.8	O6—C22—C14	123.5 (3)
C5—C6—C7	119.6 (4)	O6—C22—C21	122.1 (3)
C5—C6—H6	120.2	C14—C22—C21	114.4 (3)
C7—C6—H6	120.2	O4—N1—O5	120.4 (3)
C2—C7—C6	118.3 (4)	O4—N1—C11	118.6 (3)
C2—C7—C8	116.7 (3)	O5—N1—C11	120.9 (3)
C6—C7—C8	125.0 (4)	C12—N2—C13	125.5 (4)
C9—C8—O3	122.9 (3)	C12—N2—H2	117.3
C9—C8—C7	122.3 (3)	C13—N2—H2	117.3
O3—C8—C7	114.8 (3)	C1—O2—C2	122.3 (3)
C8—C9—C1	119.5 (3)	C12—O3—C8	119.7 (3)
C8—C9—C10	122.2 (3)	C15—O7—C16	118.5 (3)
C1—C9—C10	118.3 (3)	Cl1'—C23—Cl3'	125.6 (5)
C9—C10—C11	108.5 (3)	Cl1'—C23—Cl3	117.6 (7)
C9—C10—C14	109.8 (3)	Cl3'—C23—Cl3	55.4 (3)
C11—C10—C14	112.0 (3)	Cl1'—C23—Cl2	82.4 (5)
C9—C10—H10	108.8	Cl3'—C23—Cl2	151.9 (5)
C11—C10—H10	108.8	Cl3—C23—Cl2	112.8 (5)
C14—C10—H10	108.8	Cl1'—C23—Cl2'	118.9 (5)
N1—C11—C12	120.7 (3)	Cl3'—C23—Cl2'	114.1 (5)
N1—C11—C10	117.0 (3)	Cl3—C23—Cl2'	85.0 (6)
C12—C11—C10	122.3 (3)	Cl2—C23—Cl2'	38.2 (5)
N2—C12—O3	112.1 (3)	Cl1'—C23—Cl1	27.2 (4)
N2—C12—C11	127.7 (3)	Cl3'—C23—Cl1	99.0 (4)
O3—C12—C11	120.3 (3)	Cl3—C23—Cl1	109.5 (5)
N2—C13—H13A	109.5	Cl2—C23—Cl1	109.1 (4)
N2—C13—H13B	109.5	Cl2'—C23—Cl1	146.1 (5)
H13A—C13—H13B	109.5	Cl1'—C23—H23A	123.7
N2—C13—H13C	109.5	Cl3'—C23—H23A	60.5
H13A—C13—H13C	109.5	Cl3—C23—H23A	108.4
H13B—C13—H13C	109.5	Cl2—C23—H23A	108.4
C15—C14—C22	120.1 (3)	Cl2'—C23—H23A	94.7
C15—C14—C10	120.2 (3)	Cl1—C23—H23A	108.4
C22—C14—C10	119.6 (3)	Cl1'—C23—H23B	93.9
C14—C15—O7	124.9 (3)	Cl3'—C23—H23B	93.9
C14—C15—H15	117.6	Cl3—C23—H23B	144.5
O7—C15—H15	117.6	Cl2—C23—H23B	85.5
O7—C16—C21	121.5 (3)	Cl2'—C23—H23B	93.9
O7—C16—C17	117.2 (3)	Cl1—C23—H23B	90.9
C21—C16—C17	121.4 (4)	H23A—C23—H23B	36.2
C18—C17—C16	119.4 (4)		
			
O2—C2—C3—C4	179.3 (4)	C10—C14—C15—O7	−178.8 (3)
C7—C2—C3—C4	−0.1 (7)	O7—C16—C17—C18	178.9 (4)
C2—C3—C4—C5	−0.4 (7)	C21—C16—C17—C18	−1.4 (6)
C3—C4—C5—C6	0.1 (8)	C16—C17—C18—C19	−0.3 (7)
C4—C5—C6—C7	0.6 (7)	C17—C18—C19—C20	1.7 (6)
C3—C2—C7—C6	0.7 (6)	C17—C18—C19—Br1	−179.2 (3)
O2—C2—C7—C6	−178.5 (3)	C18—C19—C20—C21	−1.3 (6)
C3—C2—C7—C8	−178.1 (4)	Br1—C19—C20—C21	179.6 (3)
O2—C2—C7—C8	2.6 (5)	O7—C16—C21—C20	−178.6 (3)
C5—C6—C7—C2	−1.0 (6)	C17—C16—C21—C20	1.7 (6)
C5—C6—C7—C8	177.8 (4)	O7—C16—C21—C22	2.1 (5)
C2—C7—C8—C9	1.9 (5)	C17—C16—C21—C22	−177.5 (4)
C6—C7—C8—C9	−176.9 (4)	C19—C20—C21—C16	−0.4 (5)
C2—C7—C8—O3	−178.9 (3)	C19—C20—C21—C22	178.9 (3)
C6—C7—C8—O3	2.3 (5)	C15—C14—C22—O6	−176.2 (4)
O3—C8—C9—C1	175.2 (3)	C10—C14—C22—O6	2.2 (6)
C7—C8—C9—C1	−5.7 (5)	C15—C14—C22—C21	3.0 (5)
O3—C8—C9—C10	−6.7 (5)	C10—C14—C22—C21	−178.7 (3)
C7—C8—C9—C10	172.4 (3)	C16—C21—C22—O6	175.4 (4)
O1—C1—C9—C8	−175.3 (4)	C20—C21—C22—O6	−3.9 (6)
O2—C1—C9—C8	4.9 (5)	C16—C21—C22—C14	−3.8 (5)
O1—C1—C9—C10	6.6 (6)	C20—C21—C22—C14	176.9 (3)
O2—C1—C9—C10	−173.2 (3)	C12—C11—N1—O4	−176.3 (3)
C8—C9—C10—C11	19.9 (4)	C10—C11—N1—O4	0.6 (5)
C1—C9—C10—C11	−162.0 (3)	C12—C11—N1—O5	3.5 (5)
C8—C9—C10—C14	−102.7 (4)	C10—C11—N1—O5	−179.6 (3)
C1—C9—C10—C14	75.4 (4)	O3—C12—N2—C13	−1.6 (6)
C9—C10—C11—N1	161.2 (3)	C11—C12—N2—C13	179.6 (4)
C14—C10—C11—N1	−77.5 (4)	O1—C1—O2—C2	179.6 (4)
C9—C10—C11—C12	−22.0 (5)	C9—C1—O2—C2	−0.5 (5)
C14—C10—C11—C12	99.3 (4)	C3—C2—O2—C1	177.5 (4)
N1—C11—C12—N2	6.3 (6)	C7—C2—O2—C1	−3.2 (6)
C10—C11—C12—N2	−170.4 (4)	N2—C12—O3—C8	−173.6 (3)
N1—C11—C12—O3	−172.3 (3)	C11—C12—O3—C8	5.3 (5)
C10—C11—C12—O3	11.0 (5)	C9—C8—O3—C12	−7.5 (5)
C9—C10—C14—C15	−121.8 (4)	C7—C8—O3—C12	173.3 (3)
C11—C10—C14—C15	117.6 (4)	C14—C15—O7—C16	−1.5 (6)
C9—C10—C14—C22	59.9 (4)	C21—C16—O7—C15	0.6 (5)
C11—C10—C14—C22	−60.7 (4)	C17—C16—O7—C15	−179.7 (4)
C22—C14—C15—O7	−0.4 (6)		

### Hydrogen-bond geometry (Å, º)

**Table:**

*D*—H···*A*	*D*—H	H···*A*	*D*···*A*	*D*—H···*A*
N2—H2···O5	0.86	2.00	2.622 (5)	128
N2—H2···O5^i^	0.86	2.37	3.063 (5)	138
C4—H4···O7^ii^	0.93	2.59	3.383 (6)	144
C15—H15···O4^iii^	0.93	2.36	3.221 (4)	153

Symmetry codes: (i) −*x*, −*y*+1, −*z*+1; (ii) *x*, *y*, *z*−1; (iii) −*x*+1, −*y*+1, −*z*+1.
