# Supplementary figures and images for: Crystal structure of 4-(6-bromo-4-oxo-4H-chromen-3-yl)-2-methyl­amino-3-nitro­pyrano[3,2-c]chromen-5(4H)-one chloro­form monosolvate
Source: Acta Crystallogr E Crystallogr Commun. 2015 Aug 6;71(Pt 9):o648–9. doi: 10.1107/S2056989015014553 (PMC4555410; doi:10.1107/S2056989015014553)

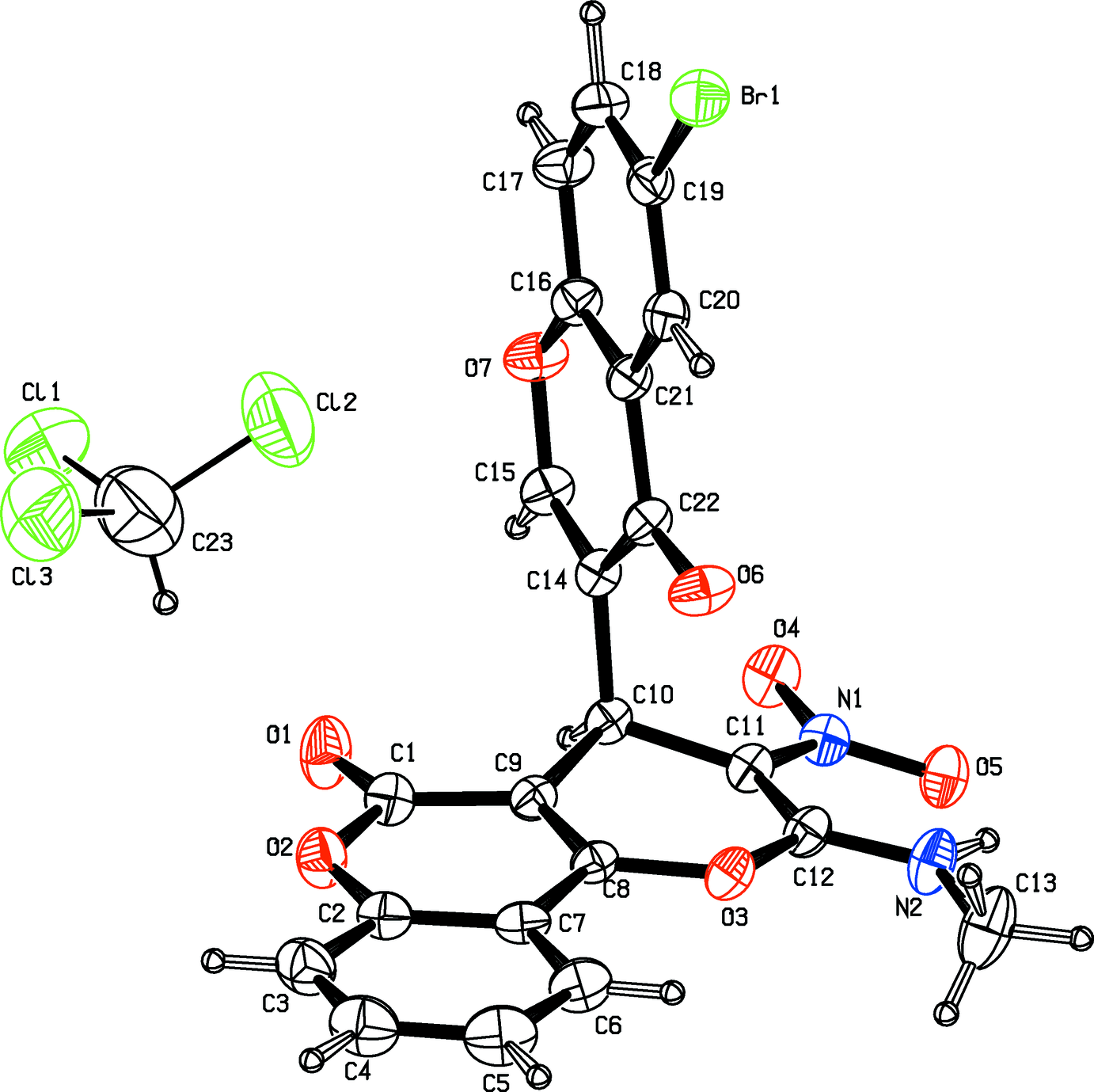

Supplement: Supplementary file 4 [file e-71-0o648-fig1.tif]

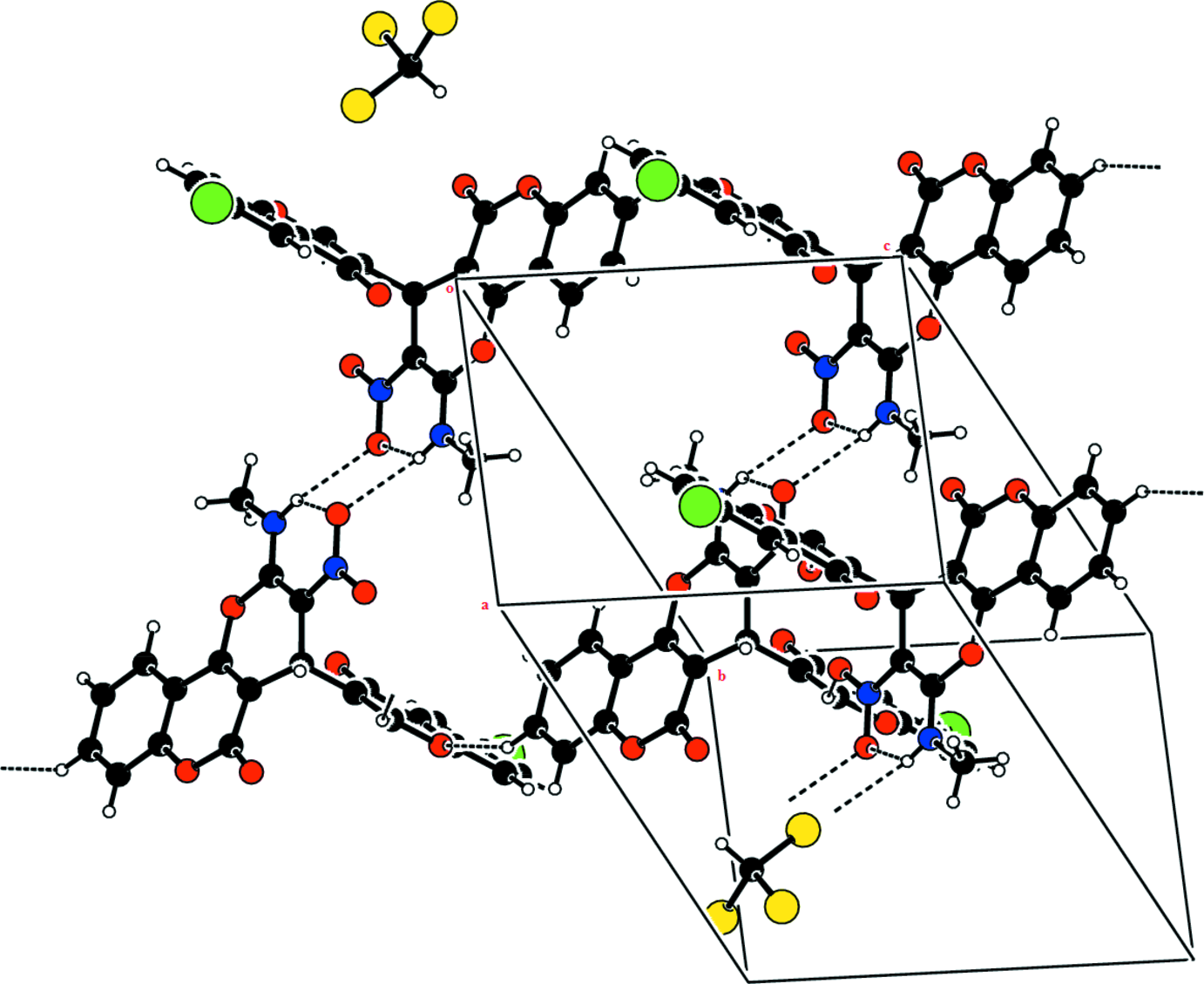

Supplement: Supplementary file 5 [file e-71-0o648-fig2.tif]
